# Low-Intensity Ultrasound Combined with Hematoporphyrin Monomethyl Ether in the Treatment of Experimental Periodontitis in Rats

**DOI:** 10.1155/2016/7156716

**Published:** 2016-11-16

**Authors:** Deshu Zhuang, Zongshan Ji, Liangjia Bi, Xiaochun Wang, Qi Zhou, Wenwu Cao

**Affiliations:** ^1^Department of Stomatology, The Fourth Affiliated Hospital, Harbin Medical University, Harbin 150001, China; ^2^Department of Cadre's Ward, The First Hospital of Harbin City, Harbin 15001, China; ^3^Condensed Matter Science and Technology Institute, Harbin Institute of Technology, Harbin 150080, China; ^4^Department of Mathematics and Materials Research Institute, The Pennsylvania State University, University Park, PA 16802, USA

## Abstract

*Objectives*. This study aims to evaluate the efficacy of hematoporphyrin monomethyl ether- (HMME-) mediated sonodynamic therapy (SDT) on experimental periodontal disease in rats.* Methods*. Periodontal disease was induced by submerging ligatures at the first maxillary molar subgingival region in forty-eight male SD rats. After 30 days, the ligatures were removed. The rats were randomly allocated into four groups; the experimental SDT group was treated through hypodermic injection of 40 *μ*g/mL HMME and 3 W/cm^2^ low-intensity ultrasound irradiation (1 MHz, 600 s). Those in control groups received 40 *μ*g/mL HMME alone (control 1 group) or 3 W/cm^2^ ultrasound irradiation alone (control 2 group) or were subjected to neither HMME nor ultrasound (control 3 group). After 10 days of treatment, all rats were euthanized, the maxilla was obtained for histological examination, and the alveolar bone level was evaluated by histometric analysis.* Results*. The control groups showed more bone loss (*P* < 0.05) after 10 days of treatment than the SDT group. There is no significant difference among the control groups (*P* > 0.05).* Conclusions*. HMME mediated SDT was an effective therapy of experimental periodontal tissue in rats.

## 1. Introduction

Periodontitis is a chronic inflammatory infection induced by some pathogenic microorganisms [1]. Most of these pathogenic bacteria are facultative or obligate anaerobes. Evidence to date indicates that Gram-negative microbes, represented by* Porphyromonas gingivalis*,* Actinobacillus actinomycetemcomitans*,* Tannerella forsythensis*, and* Fusobacterium nucleatum*, are main important periodontal pathogens [2]. In the oral cavity, the pathogens constantly proliferate. Due to the masticational function and tooth structure of human, these pathogens gathered around the gingival margin and formed dental plaque on the dental neck after being mixed with salivary polysaccharides and glycoproteins [3]. The settlement of dental plaque is very stable and difficult to wash or gargle away unless removed by using mechanical method [4]. Microbial plaque can produce lipopolysaccharide, numerous proteases, and capsular polysaccharide to trigger immune response in gingival tissue [5]. However, excessive reproduction of inflammatory cells often leads to connective tissue destruction and alveolar bone resorption [6]. Therefore, effectively restraining the dental plaque growth and periodontal inflammation progress has become the key to the treatment of periodontitis.

Removal of dental biofilm by mechanical debridement, such as scaling and root planing, and inactivation of bacteria by application of antibiotics, such as penicillin, cephalosporin, and minocycline hydrochloride, are clinical familiar treatment of periodontopathy [7]. Although mechanical debridement is available to remove the pathogenic microorganisms on the surface of the tooth root, it cannot in the long-term maintain oral cavity microecological environment and resist frequent reinfection of bacteria and fails to reach the furcation area and connective tissue which hide an amount of dental plaque [8]. In addition, repeating operation of scaling and root planing bring pain and trauma to the patient [9]. The effect of systemic or topical antibiotics is usually not completely satisfactory and might cause xerostomia and bacteria resistance during the therapeutic process [10]. Therefore, to explore an noninvasive and effective therapeutic method of periodontitis is imminent.

Sonodynamic therapy (SDT) is a relatively new therapeutic method in cancer treatment, and it has produced great progress in recent years [11, 12]. SDT is a noninvasive method utilizing the effect of low-intensity ultrasound and a sonosensitizer. Using this technique, sonosensitizer is activated by irradiation with low-intensity ultrasound of a suitable frequency, generating reactive oxygen species (ROS), such as free radicals and singlet oxygen, to kill target cells [11]. Based on the characteristic of ultrasound, SDT has many advantages over conventional periodontal treatment, such as no drug resistance, noninvasive, easy repeatability, and strong penetrating power [13]. Recent experimental studies showed that SDT can effectively kill* Staphylococcus aureus* [13], methicillin-resistant* Staphylococcus aureus* [14], and inflammatory cells, such as U937 and THP-1 [15, 16]. However, the effect of SDT using 3 W/cm^2^ low-intensity ultrasound on experimental periodontal disease in rats has not been clarified.

Experimental practice has showed that hematoporphyrin monomethyl ether (HMME) has a stable composition and low toxicity in SDT to treat disease, and HMME is also very cheap in price [15]. It consists of two positional isomers of 3-(1-methyloxyethyl)-8-(1-hydroxyethyl) deuteroporphyrin IX and 8-(1-methyloxyethyl)-3-(1-hydroxyethyl) deuteroporphyrin IX [13]. Some researches indicated that HMME can be a sonosensitizer in SDT to inhibit cancer cells, inflammatory cells, and bacteria [13, 15]. After being activated by ultrasound, HMME generated ROS to induce cell apoptosis through the mitochondrial apoptotic pathway [17]. These studies demonstrated that HMME might be a good candidate for SDT.

Our previous study showed HMME combined with 1 W/cm^2^ low-intensity ultrasound could effectively alleviate the bone loss in furcation region of the mandibular first molar with induced periodontitis through eight times of the treatment, each time for 30 min [18]. Considering the periodontal tissue of the adjacent clearance between the first and second maxillary molars was the initial and the worst part of the induced periodontitis [19], this part was treated on our present study, and the distance between the cementoenamel junction (CEJ) and the alveolar bone crest (ABC) was measured to access the level of alveolar bone. However, the therapeutic effect and the treatment time were very important on clinical therapy. On the basis of our previous study, we promoted the ultrasound intensity to 3 W/cm^2^, shortened the treatment time to 600 s, and reduced the number of therapy to 5 times, to explore the potential for SDT efficacy upon periodontitis in rats.

Considering the seemingly promising therapeutic method of SDT, we hypothesized that the use of SDT may suppress the alveolar bone resorption. The aim of this study was to evaluate the efficacy of HMME-mediated SDT on experimental periodontal disease in rats.

## 2. Materials and Methods

### 2.1. Sonosensitizer and Ultrasound System

**Figure 1:**
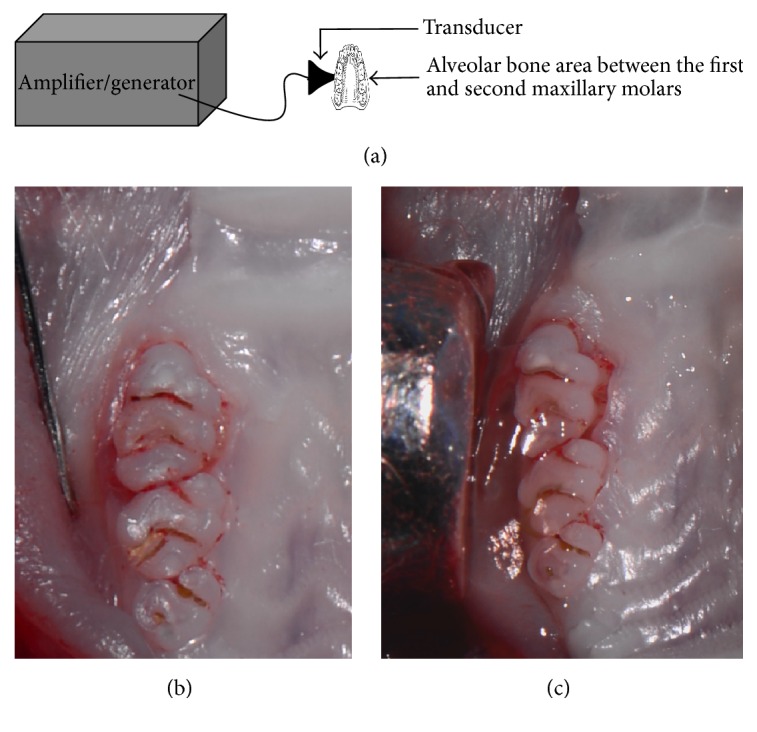
Schematic diagram of the SDT system. Notes: (a) schematic diagram of the ultrasound system. (b) Photograph in vivo of the injection site of HMME. (c) Photograph in vivo of the transducer in action.

We prepared a HMME solution by diluting 500 *μ*g/mL HMME sterile solution (Xianhui Pharmaceutical co., Shanghai, People's Republic of China) in sterile deionized water to obtain final concentration of 40 *μ*g/mL and immediately kept in the dark. The configured HMME solution was kept in refrigerator at 4°C before use. The ultrasound equipment described in Figure 1(a), including ultrasonic generator, power amplifier, and ultrasonic transducer, was assembled and provided by Harbin Institute of Technology (Harbin, People's Republic of China). The ultrasonic transducer described in Figure 1(c), with a ultrasound frequency of 1.0 MHz, a pulse repetition frequency of 100 Hz, and a duty factor of 10%, was used to illuminate the alveolar bone area between the first and second maxillary molars. The jut of the transducer has a diameter of 6 mm. Before ultrasound irradiation, the ultrasonic transducer, expressed as *I*_SPTP_ (spatial peak/temporal peak), was adjusted to 3 W/cm^2^ and the irradiation area was connected by medical ultrasonic coupling agent. The intensity of ultrasound was carefully calibrated with a hydrophone (Onda Corp., Sunnyvale, CA, USA) in degassed distilled water, which measured 6 mm between the jut of the transducer and hydrophone.

### 2.2. Animal Model Produced

Forty-eight male SD rats were obtained from the animal experimental center of the Second Affiliated Hospital of Harbin Medical University (Harbin, People's Republic of China). All rats weight 200–250 g at an average of 45 days' age group. The rats were kept in polyethylene plastic cages and were fed with rat feedstuff and water ad libitum. All rats were kept to acclimatize to the housing conditions for 5 days before the operative procedures. Animal care and experimental procedures were carried out in accordance with the National Institutes of Health guide for the care and use of Laboratory animals (NIH Publications Number 8023, revised 1978).

Rats were anesthetized with 10% chloral hydrate (5 mL/kg) via intraperitoneal injection. 1% isoflurane was maintained during the anesthesia process. The following process was operated by the same experienced operator (Deshu Zhuang): To induce experimental periodontitis, the right side of maxillary first molars of the rat was immediately selected to obtain cotton ligatures around the subgingival position. The contralateral maxillary first molars of the rat received no treatment. After 30 days, the cotton ligatures of maxillary first right molars were removed. The forty-eight rats were randomly allocated, using a computer-generated table, into four groups (12 animals in each group); the experimental SDT group was treated through hypodermic injection of 40 *μ*g/mL HMME and 3 W/cm^2^ low-intensity ultrasound irradiation (1 MHz, 600 s). The infection sites of each rat, shown in Figure 1(b), are the alveolar bone area between the first and second maxillary molars. Those in control groups received 40 *μ*g/mL HMME alone (control 1 group) or 3 W/cm^2^ ultrasound irradiation alone (control 2 group) or were subjected to neither HMME nor ultrasound (control 3 group).

### 2.3. Sonodynamic Therapy

The rats were anesthetized before treatment, and the HMME solution was injected into the periodontal tissue. Then the rats were placed in the dark for 90 min. After that, the experimental inflammatory sites of rats were radiated by ultrasound for 600 s (SDT group). The effect of sonosensitizer alone was tested by injection of 40 *μ*g/mL HMME solution at the same time as the SDT group, without the ultrasound treatment (control 1 group). To explore the effect of the ultrasound alone, the HMME solution was replaced with 0.9% sterilizing saline, and ultrasound radiation was the same as the SDT group (control 2 group). The last group of rats received 0.9% sterilizing saline alone, with no ultrasound treatment (control 3 group). During the SDT procedure, the temperature on the jut of the transducer almost did not change measured by a thermocouple. The animals in each group were treated every other day, totaling 5 times.

### 2.4. Histological Evaluation

After these respective treatments, the rats were sacrificed immediately. Their maxillas were removed and demineralized in EDTA solution for 40 days. Next, the samples were dehydrated with serial alcohol and then mounted in paraffin. Step serial sections, 6 *μ*m thick, were cut in a mesiodistal direction with hematoxylin and eosin (H&E).

The linear distance from the CEJ to ABC in the alveolar bone area between the first and second maxillary molars was measured by light microscopy and recorded by digital camera that utilized an image analysis software (Image Tool, Harbin Medical University). The measurements were made by an examiner who was masked to the samples (Zongshan Ji). After excluding the first and the last sections where the alveolar bone area was evident, ten equidistant sections of each specimen block were selected [19].

### 2.5. Statistical Analysis

All measured values were normally distributed. The histometric data were statistically analyzed with the PC software SPSS 20.0 and expressed as mean ± standard error. Differences of interblock were performed with a one-way analysis of variance (ANOVA), considering statistically significant at *P* < 0.05.

## 3. Results

**Figure 2:**
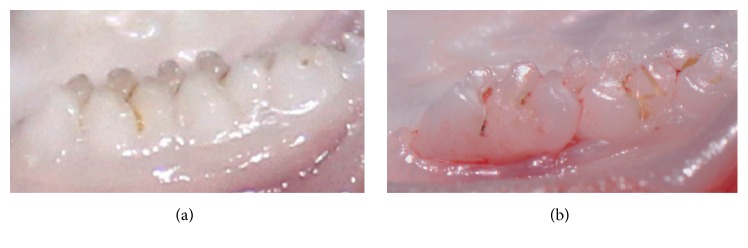
Photographs of rat gingiva. Notes: (a) healthy rat with no treatment; (b) 30 days after induction on experimental periodontitis.

Comparison of the gross appearance of the formation process of periodontitis is shown in Figure 2. Healthy gums were found in the rats with no ligatures (Figure 2(a)). After 30 days after induction on experimental periodontitis (Figure 2(b)), the periodontal tissue became red and swollen, bled easily, and had some attachment loss.

**Figure 3:**
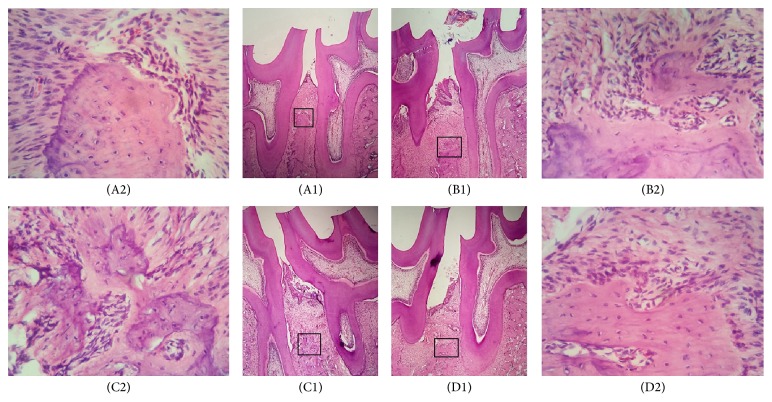
Photomicrographs of bone tissue in the alveolar bone area between the first and second maxillary molars with induced periodontitis. Notes: (A1) SDT group at 10 days. (A2) Areas of thick bone trabecula without signs of resorption in the SDT group at 10 days. (B1) Control 1 group at 10 days. (B2) Areas of bone resorption with thin bone trabecula and disorganized connective tissue in the control 1 group at 10 days. (C1) Control 2 group at 10 days. (C2) Areas of bone resorption with thin bone trabecula and disorganized connective tissue in the control 2 group at 10 days. (D1) Control 3 group at 10 days. (D2) Areas of bone resorption with thin bone trabecula and disorganized connective tissue in the control 3 group at 10 days. Hematoxylin and eosin staining; original magnification for (A1), (B1), (C1), and (D1) was ×4 and for (A2), (B2), (C2), and (D2) was ×40.

Among the control 1 group (Figures 3(B1) and 3(B2)), control 2 group (Figures 3(C1) and 3(C2)), and control 3 group (Figures 3(D1) and 3(D2)) after 10 days of treatment, the gingival connective tissue showed an intense inflammatory infiltrate throughout the periodontal ligament, and most of the inflammatory cells were polynuclear leukocytes. The connective tissue displayed a small number of fibroblasts, and the bone tissue exhibited an area of resorption. In the SDT group (Figures 3(A1) and 3(A2)), at 10 days after treatment, the connective tissue was well organized with no inflammatory infiltrate. The bone tissue did not show resorption in all specimens.

**Figure 4:**
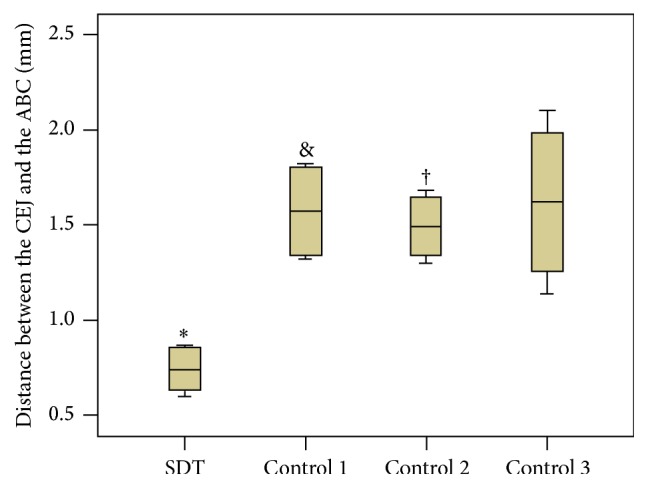
Mean ± standard deviation of the data of the distance between the CEJ and ABC (mm) of the alveolar bone area between the first and second maxillary molars in each group. Notes: ^*∗*^compared with the control 3 group (*P* < 0.05; ANOVA). ^&^Compared with the control 3 group (*P* > 0.05; ANOVA). ^†^Compared with the control 3 group (*P* = 0.068; ANOVA). ANOVA, analysis of variance.

Histometric analysis showed a significant difference between groups in alveolar bone loss (Figure 4). Compared with the SDT group, all control groups showed significantly more alveolar bone loss (*P* < 0.05). Among the three control groups, there was no statistical difference between control 1 group and control 3 group (*P* > 0.05). Although no significant difference was found between control 2 group and control 3 group (*P* > 0.05), it is worth mentioning that, at 10 days after treatment, a minor difference in alveolar bone loss was obtained between the control 2 and control 3 groups (*P* = 0.068).

## 4. Discussion

In our study, the SD rats were used to establish the experimental periodontitis model by embed cotton ligatures in the subgingival position. After 30 days, the periodontal tissue became red and swollen and had some attachment loss. Pathological results showed that there were a lot of inflammatory cells infiltration, seldom fibroblasts of connective tissue disorganization, and bone loss in coronal furcation. These clinical and pathological alterations are in line with those of other experimental periodontitis model establishment reported by other investigators [20, 21].

HMME mediated SDT has found its success as a treatment in vitro [15]. In recent studies, Sun et al. [22] demonstrated that SDT with HMME induced the apoptosis of endometrial cancer cells. Liu et al. [23] demonstrated that HMME-SDT action markedly induced the apoptosis of MG-63 cells. Based on the strong penetrating power of ultrasound, the researchers focused on the exploration of SDT in vivo. Many results showed that SDT appeared to be efficient in the treatment of tumor tissue, such as mouse S-180 sarcoma and tongue squamous carcinoma [24, 25]. In our study, the effect of HMME mediated SDT on experimental periodontal disease was investigated in rats.

The selective anti-inflammatory effect of SDT is based on selective uptake of a sonosensitizer by inflammation [15]. Sonosensitizers are molecules that are highly sensitive and selective. The metabolically active cells in tissue will be as a target and accumulated. But the sensitizers have noncytotoxicity in a certain concentration range and do not damage the surrounding normal tissues [24, 25]. HMME possess a stable wavelength and a short incubation time, compared with hematoporphyrin derivative (HPD) [13]. Previous studies have indicated that the HMME concentration of 40 *μ*g/mL activated by laser reduced the growth of THP-1 cells [26]. And more than 95% of* Staphylococcus aureus* were killed by SDT when the HMME concentration is 50 *μ*g/mL [18]. In the present study, we chose HMME 40 *μ*g/mL as sonosensitizers in SDT. In contrast with control 1 group, there was no therapeutic efficiency on experimental periodontitis with 40 *μ*g/mL HMME alone in control 2 group (*P* > 0.05). And this result is in agreement with other reports [18, 26].

Although there was no significant difference compared with control 2 group and control 3 group (*P* > 0.05), the histometric analyses demonstrated that, at 10 days after treatment, the alveolar bone loss level of control 2 group was slightly less than control 3 groups (*P* = 0.068). This may be due to the ability in promoting tissue recovery and healing of low-intensity ultrasound [25]. As several studies mentioned, the low-intensity ultrasound could promote angiogenesis and cell proliferation, accelerate the collagen synthesis, and resist inflammation [27, 28], although the ultrasound alone treatment had no difference compared with control 3 group after 10 days of treatment in our research. A significant effect may be obtained after a longer treatment time, more than 10 days.

The results for periodontal specimens in the SDT group, in which the sonosensitizing drug was associated the ultrasound, revealed statistically significant differences in alveolar bone loss after 10 days of treatment (*P* < 0.05). Insight into these mechanisms gained from SDT studies has established that the activation of sonosensitizer was via sonoluminescence and/or sonochemistry [12–18]. From the sonoluminescence perspective, the flashes of light produced during bubble collapse in acoustic cavitation excite the sonosensitizer to produce the reactive oxygen species (ROS) [15–17]. From the sonochemistry perspective, the sonosensitizer-derived ROS are produced either by direct pyrolysis or via reactions with other ROS formed by the pyrolysis of water. These ROS then react with dissolved oxygen to form other ROS, including peroxyl and alkoxyl radicals, which are able to initiate damage to critical cellular sites [29]. Although the mechanism of SDT is poorly understood, there is general agreement that SDT can generate singlet oxygen and other ROS, which are responsible for irreversible damage on cell cytoplasmic membrane, mitochondrial proteins, and cell membrane permeabilization [30]. This probably explained why the beneficial effect of SDT is more pronounced than other treatments in periodontal disease in rats.

## 5. Conclusions

Our study may provide useful information for applying SDT in experimental periodontal disease in rats. However, further studies on the effect of bacterial biofilm and inflammatory cells by SDT are greatly needed. Compared with the no-treatment group, SDT could effectively alleviate the inflammatory reactions in the periodontal tissue with no detectable damage. We conclude that HMME mediated SDT is an noninvasive method and has the potential to suppress the alveolar bone resorption. These encouraging results suggest that SDT may provide a promising treatment against periodontal disease.
